# Intercultural adjustment of internationally mobile academics working in Thailand

**DOI:** 10.1007/s10734-022-00846-4

**Published:** 2022-04-08

**Authors:** Alina Schartner, Tony Johnstone Young, Navaporn Snodin

**Affiliations:** School of Education, Communication and Language Sciences (ECLS), Newcastle University, Newcastle upon Tyne, NE1 7RU UK

**Keywords:** Academic migration, Internationalisation, Adjustment, Intercultural transitions, International mobility, Internationally mobile academics

## Abstract

Non-nationals constitute up to a quarter of the academic staff workforce of Higher Education Institutions (HEIs) worldwide. Their motivations to ‘work abroad’, and their experiences of doing so, are, however, under-researched, especially where migration is to or within the global South. We report a study conducted among internationally mobile academics from a variety of countries working in Thailand. At policy level, Thailand aspires to increase the numbers of international staff and students in its HEIs, but with mixed success in terms of recruitment levels and the reported quality of the experience among these migrants. Conceptually, our study drew on a framework of intercultural adjustment defined as a multidimensional construct comprising psychological, sociocultural and professional/work aspects of the experience. Semi-structured focus groups were conducted to explore experiences of working in Thailand. Thematic analysis of findings showed that reported experiences mainly fell under the category of professional/work aspects of intercultural adjustment, although sociocultural issues were also important. The reported quality of experiences was mixed and generally more negative than positive in the key areas of professional and work adjustment. Specific challenges highlighted by participants related to issues with the work environment, most especially lingual-cultural problems and, crucially, a lack of secure status. We detail and discuss these findings and present recommendations for policy makers and HEIs, aspirant academic migrants, and for future research into global academic migration, particular as it relates to Thailand and other destinations for academic migration.

## Introduction and literature review

The migration of internationally mobile academics is a growing, global higher education (HE) phenomenon. Although accurate statistics are not available, there is no doubt that this group plays a central role in the global academic workforce (Teichler, 2017; Mihut et al., 2017). Some estimates suggest that it is not uncommon for some universities to recruit a quarter or more of their academic staff from overseas (Trembath, 2016). Others have found significant global variations in international staff numbers ranging from 26% in the UK to around 5% in Japan, for example (De Wit et al., 2015). Whilst international student mobility is now a firmly established research area (see Schartner & Young, 2016, 2020), relatively little is still known about the experiences of internationally mobile academic staff (Mihut et al., 2017). An especially under-researched group are academics who choose to migrate outside the global Northern, most usually Anglophone HE systems (Mason & Rawlings-Sanaei, 2014; Richardson &Wong, 2018; Burford et al., 2019). Where this kind of research is being conducted, it is more commonly related to short-term mobility (sojourning) as opposed to longer-term mobility, better characterised as academic migration (Mihut et al., 2017).

Despite their prevalence, internationally mobile academic staff are a hard to define population (Rumbley & de Wit, 2017). Some countries apply citizenship or visa categories to define who is ‘international’, while others leave it up to individual institutions (Altbach & Yudkevich, 2017). Several terms abound in the literature, including ‘expatriate academics’ (Trembath, 2016; Ramalu and Subramaniam, 2019), ‘foreign/international faculty’ (Altbach & Yudkevich, 2017), ‘international academics’ (Wan and Morshidi, 2018), and ‘internationally mobile academics’ (Burford et al., 2019). In this paper we adopt the latter term as we find it the most inclusive. Following Altbach and Yudkevich (2017: 1), we define this group as ‘academics who hold appointments in countries where they were not born and/or where they did not receive their first postsecondary degree.’ Whilst Altbach and Yudkevich include solely those in permanent, full-time appointments in their definition, we apply a slightly broader conceptualisation which also includes academic researchers and/or teachers on fixed-term contracts at postdoctoral level and above. Our working definition of the participant group in the study reported here was therefore ‘People working as academics in a Thai Higher Education Institution (HEI) whose countries of origin is not Thailand and whose education was primarily received in a country other than Thailand’.

Motivations for academics to seek out employment abroad include both ‘push’ and ‘pull’ factors (see Kuzhabekova & Lee, 2018). The former might include HE-specific factors such as poor working conditions, lack of academic freedom or research funding, and limited career opportunities in the home country (Mihut et al., 2017). More broadly, this might also include ethnic persecution, political instability, human rights abuses, and personal safety concerns (Khan, 2021; Kline, 2003; Welch, 2008, 2012). The latter may include perceived financial gains, better career progression, interest or personal ties in the host country or region, the prestige of a particular institution, and a desire for international experiences more broadly (see Froese, 2012; Selmer & Lauring, 2012, 2013; Rumbley & de Wit, 2017; Wu & Huang, 2018).

There are several reasons why universities recruit academic staff from overseas. These are all, to a greater or lesser extent, influenced by local governmental policies designed to increase the ‘international’ nature of their HE provision, and a perceived need at both institutional and national levels to compete in the neo-liberal, international educational ‘marketplace’ (Schartner & Young, 2020). The first reason is, in an increasingly competitive and globalised HE landscape universities are more likely to look beyond their own borders to attract the best possible talent (Burford et al., 2019; Trembath, 2016), often in an effort to boost research productivity and to increase an institution’s position in international league tables (Burford et al., 2019). Secondly, in an environment where many universities increasingly proclaim their ‘international’ status (see Robson et al., 2018), the recruitment of international staff is often a central part of institutional internationalisation strategies as ratios of international staff are a common proxy for the degree of internationalisation ‘achieved’ (Altbach & Yudkevich, 2017; Ramalu & Subramaniam, 2019). In some non-English speaking countries international staff are also key drivers of ‘Englishization’ and are recruited to increase the number of courses taught in English, another key internationalisation indicator in some contexts (Altbach & Yudkevich, 2017). Thirdly, the rise of the knowledge-based economy and rapid expansions of national HE systems have resulted in a shortage of qualified local staff in some countries, in particular in the Middle East (see Austin et al., 2014), and academics are often recruited from overseas to fill these gaps (Altbach & Yudkevich, 2017). Finally, there are now more opportunities for mobility at the regional and global level than ever before, not least due to favourable migration paths created by transnational organisations such as the European Union (EU), Mercosur, or the Association of Southeast Asian Nations (ASEAN) (Mihut et al., 2017), making it less likely for academics to remain committed to a single university or country (Trembath, 2016). Despite the COVID-19 pandemic, it seems likely that all of these motivations to recruit non-local academic staff will pertain into the medium term at least (Schartner & Young, 2020), although we acknowledge that rationales and opportunities for international recruitment may vary considerably between research-intensive universities and smaller, less well resourced, institutions whose ‘brand’ is perhaps not as well established (cf. James-MacEachern, 2018).

However, global migration flows of internationally mobile academics have been found to be uneven, with countries on the global periphery often experiencing a net loss of academics (Bauder et al., 2018). The flow is generally from poorer, less developed countries to richer ones which offer better financial and career-enhancement opportunities. This is mirrored in existing studies which focus, by and large, on the experiences of internationally mobile academics in highly developed parts of the globe (Richardson & Wong, 2018), in particular Anglophone countries such as the United Kingdom (e.g. Luxon & Peelo, 2009; Gimenez & Morgan, 2014; Walker, 2015), the United States (e.g. Kim et al., 2011; Lawrence et al., 2014), and Australia (e.g. Balasooriya et al., 2014), as well as Northern Europe (e.g. Selmer & Lauring, 2010, 2011). However, there is increasing scholarly interest in the experiences of academics who choose to migrate to emerging economies in the Middle East (e.g. Austin et al., 2014), East Asia (e.g. Froese, 2012; Cai & Hall, 2016; Wu & Huang, 2018), and South East Asia (e.g. Lauring & Selmer, 2014; Ramalu & Subramaniam, 2019). Among the latter group of countries, Thailand has in recent years emerged as an increasingly popular destination for internationally mobile academics, but there is little research to date on the experiences of these individuals (for notable exceptions see Snodin et al., 2021; Hoy, 2009 and Burford et al., 2020) with most studies on transnational mobility in the Thai context focussing on student sojourners (e.g. Srisakda, 2018; Snodin, 2019).

Thailand is aspiring to become a regional HE hub (Snodin, 2019), and the Office of the Higher Education Commission (OHEC) is now actively promoting the internationalisation of Thai HE. To date, these efforts have largely focused on regional student mobility, for example through exchange programmes such as the ASEAN International Mobility for Students (AIMS) scheme, but recent efforts have been more strategically aimed at creating an international environment from which all students and staff can benefit. The country’s 20-year Education Strategic Plan issued in 2017 sets out to improve the competitiveness of Thai HE institutions, their positions in global rankings, as well as their research outputs (QAA, 2019).

HE in Thailand has seen a rapid expansion over the past decades with now 156 officially recognised HE institutions in the country, hosting over 2 million students in 2015 (Michael & Trines, 2018; QAA, 2019). Although not traditionally a major sending country of international students compared to its neighbours such as China and Vietnam (Michael & Trines, 2018), it is a popular regional destination for incoming students with an increase in international student numbers from 1,882 in 1999 to 20,309 in 2012 (ibid.). Thailand has also seen an increase in English-medium degree programmes, of which there were 1,044 in 2015, and joint-degree schemes with foreign universities, of which there were 159 in 2013 (Michael & Trines, 2018). Numbers of international academics teaching and researching in the country are much more difficult to obtain as statistics are not routinely collected by OHEC (Snodin, 2019). According to the latest available figures from the Thai Department of Employment, the number of foreign residents employed in Thai higher education institutions increased by 66.3%, from 15,858 in 2010 to 26,371 in 2018 (Department of Employment, 2018). The latest number at the time of writing this paper is 33,054, of which the majority were from the Philippines, the UK, the US and China (Department of Employment, 2019), so the effects of the COVID-19 pandemic and the subsequent global economic slowdown in 2020 and 2021 are not yet known.

**Fig. 1:**
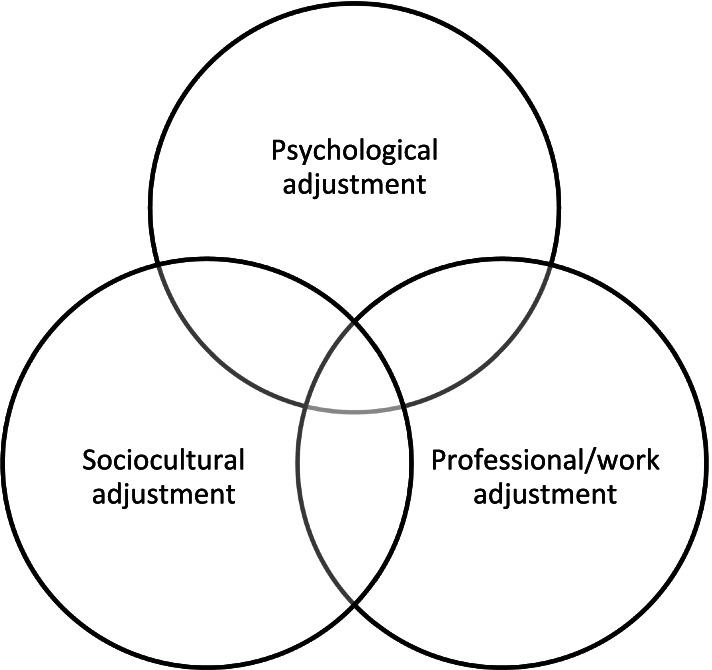
Conceptual Framework, adapted from Schartner and Young (2016, 2020)

In this paper we focus on the intercultural adjustment of internationally mobile academics in Thailand. The burgeoning literature on international student adjustment commonly frames ‘adjustment’ as a dynamic process involving interrelated psychological, sociocultural and academic aspects (Schartner & Young, Fig. 1 below). Psychological adjustment typically refers to affective responses to the host environment including subjective wellbeing and satisfaction with life (Ward et al., 2001). Sociocultural adjustment focuses on behavioural factors associated with effective and confident performance in the host environment (Furnham and Bochner, 1986). The third domain, adjustment to academic study, is less relevant to internationally mobile academics. Instead, professional/work-related adjustment seems more pertinent for this group (cf. Selmer, 2000).

While studies on international student mobility and intercultural adjustment are burgeoning (see Schartner and Young, 2020 for a recent review), research on the adjustment of academic staff working overseas is still limited (Selmer & Lauring, 2013; Richardson & Wong, 2018). Nonetheless, there is some evidence suggesting that internationally mobile staff are often less satisfied in the work environment than their domestic colleagues (e.g. Kim et al., 2012). In a study of internationally mobile academics in Malaysia, Richardson and Wong (2018) found that most adjusted well socially but reported disappointment with work-related issues. Mixed findings were reported in a study conducted in South Korea by Froese (2012) who found that while most respondents adjusted well to the new environment, many reported difficulties with housing and food in particular. There is also some evidence that internationally mobile academics may feel distant or excluded from the host ‘culture’ (Richardson & McKenna, 2006).

Adjustment challenges commonly identified in the literature include language barriers, in particular where the local language is not English (Danisman, 2017), which may prevent successful integration into the academic and social life of the institution and can negatively affect career progression in the host country (Altbach & Yudkevich, 2017; Cai & Hall, 2016). Navigating bureaucracy can also stifle adjustment as bureaucratic procedures related to work permits, visas, and security can be highly complex (Altbach & Yudkevich, 2017). Relationships with local colleagues may also be challenging as variations in salary, often reflected in higher pay for overseas staff, can lead to social tensions between international staff and their domestic colleagues (Altbach & Yudkevich, 2017). On the other hand, there is some evidence of workplace inequalities (e.g. Romanowski & Nasser, 2015) with overseas academic staff being denied tenure-track positions and roles in academic governance, and often carrying heavier teaching loads than their local counterparts (see Altbach & Yudkevich, 2017). Other studies report restricted promotion opportunities offered to overseas academics and lack of access to government research funding (Richardson & Wong, 2018). Internationally mobile academics also face logistical challenges such as finding suitable accommodation, locating appropriate schools for children and possibly also identifying employment opportunities for partners and spouses (Mihut et al., 2017).

On a professional level, challenges might include adjusting to a new academic culture and a new national HE system, as well as differences in terms of workload expectations and behaviours of students (Mihut et al., 2017). Some research has identified a lack of institutional support for non-domestic academic staff (e.g. Foote et al., 2008). A recent study of policy texts in the Thai HE context suggests that international academics may be perceived as a threat to local culture, values and security (Burford et al., 2019).

Factors that might ease adjustment to life and work for internationally mobile academics have also been identified in the literature, including degree of ‘cultural distance’ between home and host country (Isakovic & Forseth Whitman, 2013; Wilkins & Neri, 2019), clarity of work roles and responsibilities (Selmer & Lauring, 2011), and individual characteristics such as emotional resilience (Lauring & Selmer, 2015). It is important not to position internationally mobile academics as passive and powerless ‘objects’ of adjustment, but rather view them as active agents who respond to emerging circumstances in the host environment rationally and intentionally (cf. Tran & Vu, 2018).

## Data collection and analysis

In light of this literature review, the overarching research question for this study was as follows:

- What are the adjustment experiences of internationally mobile staff working in Thai higher education?

This question was informed conceptually by the Integrated Model of International Student Adjustment and Adaptation, developed by Schartner and Young (2016, 2020, Fig. 1 below), and defines intercultural adjustment as a multi-dimensional construct comprising psychological, sociocultural as well as professional/work aspects.

We acknowledge that the model is based on literature and research on international student sojourners; however we see this framework as also highly relevant to the experiences of internationally mobile academic staff (see Snodin et al., 2021).

As such, our enquiry was further guided by three related sub-questions:

1. How do internationally mobile academic staff experience their psychological adjustment?
2. How do internationally mobile academic staff experience their sociocultural adjustment?
3. How do internationally mobile academic staff experience their professional/work adjustment?

Data was collected through a series of focus groups with internationally mobile academics working in different universities across Thailand. We felt that interactions generated in a group setting would provide richer and more nuanced data not easily accessible through one-to-one interviews (Sim, 1998). In total, 17 participants took part in one of three focus groups, two conducted in the capital Bangkok and one conducted in the northern part of the country. Participants came from a variety of disciplinary backgrounds including business, economics, engineering, language teaching, physics and mathematics, and had various experience levels ranging from 2 years to 40 years, thus allowing us to capture a broad range of experience levels among our participants. They were recruited on a convenience snowball sampling basis (Bryman, 2012), and initial contact was made through the professional networks of the researchers. There was a mix of nationalities, but most came from North America or Europe (see Table 1 for demographics).

**Table 1:** Focus group demographics

Participant	Gender	Nationality
Focus Group 1 (BKK)		
P1	Male	German
P2	Female	Philippina/Swiss
P3	Male	Spanish
P4	Male	Canadian
P5	Male	UK
P6	Male	UK
Focus Group 2 (BKK)		
P1	Female	Spanish
P2	Male	USA
P3	Male	Australian
P4	Male	UK
P5	Male	German
Focus Group 3 (North)		
P1	Male	USA
P2	Female	USA
P3	Female	Jamaican
P4	Male	USA
P5	Male	UK
P6	Male	French

All focus groups took place in English and were facilitated by a moderator who was one of the co-authors of this paper, had had no professional relationship with the participants and who was not employed by any of the institutions represented. The focus groups lasted between 01:38 and 02:25 h. There was also a note-taker present and all focus groups were audio recorded with permission. An interview guide (Table 2, below) was used to facilitate the focus groups. It included a series of open prompts intended to stimulate discussion. The questions deliberately did not probe specific aspects of adjustment but rather asked participants to reflect on their experiences of living and working in Thailand more generally. This was important as we did not want to ‘lead’ participants or focus the discussion on any one particular aspect. Instead, we were interested in issues of salience as raised by the participants.

**Table 2:** Focus group prompts

• Why did you decide to come to Thailand to work? Why did you choose this particular university? • What were your initial impressions of the city and of the university? • How are things going here for you now? • What do you hope to do in the future?	

Recordings were transcribed verbatim and fully anonymised. Transcription resulted in a total written corpus of 78,432 words. The transcripts were then analysed thematically (Braun & Clarke, 2006) with the analysis guided by the conceptual framework put forward by the authors (Fig. 1). Coding was conducted by two of the authors, initially independently, and then agreed by all three authors after discussion of any contentious issues. The transcripts were read repeatedly and in a first analytical step relevant comments were sorted into four broad analytical categories, guided by the foci in our research questions and by our conceptual framework (Fig. 1), with three intersecting adjustment domains: (1) psychological adjustment, (2) sociocultural adjustment, (3) professional/work adjustment, and, for content that did not fall neatly into one of these categories, (4) other.

Each comment under each category was then further analysed for content and assigned a label or ‘node’: positive, negative, and problematizing (Young & Schartner, 2014, Schartner, 2015, see Table 3 for examples). We labelled a comment as ‘problematizing’ if it identified an aspect of living and working in Thailand as problematic while not overtly describing a negative experience or exhibiting a negative orientation (cf. Young & Schartner, 2014). The specific choice of words helped us to distinguish between ‘problematizing’ and ‘negative’ comments. For instance, if the participant indicated a clear position or used strong wording (e.g. ‘The work permit visa…I have nightmares about it.’), we classified the comment as ‘negative’. In contrast, if the participant used more subtle language or did not indicate a negative orientation per se (e.g. ‘There are a lot of unwritten rules here’), we classified the statement as ‘problematizing’.

In a third and final analytical step, content under these three nodes was analysed for emerging themes which were not directly addressed by our research questions, but which were nevertheless salient to our participants. This process allowed us to apply an analytical framework whilst also generating a collection of emerging sub-themes. In this sense the analysis was ‘abductive’ as the empirical data and theory were considered together (Gioia et al., 2013). We felt that this would give an idea of general adjustment trajectories whilst also capturing the particularity for individual participants (see Young & Schartner, 2014). At the end of this process all three authors compared notes and agreed final themes. One of the researchers was a Thai academic based in Thailand at the time of data-collection. This was important to the analysis and provided unique insights into the national context shaping the Thai HE system, including policies and cultural norms (see also Austin et al., 2014).

Key themes are presented below alongside relevant data excerpts and demographic labels (e.g. FG1 P1 = Focus Group 1, Participant 1).

**Table 3:** Analytical framework and examples

Analytical category	Example comment
Positive comment	‘I was able to work out my ideas when coming to Thailand. It was very good academic freedom.’
Negative comment	‘The work permit visa…I have nightmares about it.’
Problematizing comment	‘One big difference I’ve found between my experiences here and my experience in Western universities is basically academic disagreements here tend to be personal also.’

## Findings

### Psychological adjustment

The overall impression from the data was that there was, perhaps surprisingly, very little comment on psychological adjustment (see Table 4). There was only one participant who explicitly mentioned factors that impacted on his wellbeing, describing his first few years in Thailand as ‘very unhappy socially’ and experiencing ‘severe culture shock’.

**Table 4:** Number of comments/exchanges per analytical category

	Psychological adjustment	Sociocultural adjustment	Work adjustment	Other
Positive	0	8	18	1
Negative	1	0	40	1
Problematising	0	4	83	11
Total	1	12	141	13

### Sociocultural adjustment

There were also relatively few comments on sociocultural adjustment, although clearly the work environment to some extent forms part of the sociocultural environment (see 4.3). Where participants did comment on their sociocultural adjustment, this was mainly positive such as for example opportunities to mix with Thais outside the university context. The climate was also highlighted as a positive factor and an incentive to settle in Thailand.

*‘Here every day’s summer so I never miss it I can I mean there’s positives to being in Thailand they’re more personal they’re not career in that sense certainly not through the university.’ (FG1, P5).*

Life in Thailand was, by and large, described as ‘easy’ and the majority of participants appeared to settle well into their lives in Thailand with nobody reporting any noteworthy challenges in terms of ‘fitting in’. Most seemed satisfied with their lives in Thailand from the very beginning which was evident in comments such as ‘It’s very vibrant’ (FG2, P1), ‘I liked it. It was a very comfortable non-stressful atmosphere.’ (FG3, P1) and ‘It’s just so easy to live here.’ (FG2, P4).

*‘…as soon as I came to Thailand that feeling of I’m welcomed here and I really feel like I’m at home it’s just a feeling that is very difficult to describe but it makes you feel very very good.’ (FG2, P1).*

Explicit positive references to specific aspects of Thai culture were rare, but one participant highlighted the ‘very enjoyable Sanuk^1^ factor’ (FG1, P2).

There was some discussion about the behaviour of other international academics, with some acknowledgement of an apparent lack of understanding of Thai cultural norms and values associated with, for example, dress and ways of working.

*‘They don’t try to really understand the Thai culture which is a strong element of the the Thai life.’ (FG3, P6).*

None of the participants had received any form of cultural orientation or training prior to taking up their appointments. There was awareness that this type of preparation was available to international students but very much lacking for incoming staff, resulting in some participants still struggling to understand aspects of Thai culture several years into their stay in Thailand.

*‘I still go around and after being in Thailand for 2 and half years I’m still not sure the different levels of wai* 2 *.’ (FG3, P1).*

There was some acknowledgement that certain aspects of Thai culture were difficult to comprehend for people from overseas as illustrated in this brief exchange from FG3:

- *P5: …there’s a lot of unwritten rules there’s rules that*
- *P6: tacit rules yeah yeah yeah that most of foreigners don’t understand…*

Examples of these tacit rules included, for example, shaking hands and stepping on banknotes and coins. Participants also reported struggling to decode certain cultural markers such as smiles.

*‘…there’s like 30 different smiles in Thailand so it’s learning the different cues and trying to figure it out…’ (FG3, P2).*

### Work adjustment

With a total of 141 comments (Table 4), most talk generated by the focus group prompts revolved around the notion of ‘work adjustment’, perhaps not surprising given the status of the participants as overseas academics and the centrality of work as a key motivational factor in their decision to move abroad.

The majority of positive comments on work adjustment centred around students and classroom issues (8 comments), followed by comments on the research landscape (4), relationships with colleagues (2) and opportunities for extra work (2). Negative comments centred around immigration and visas (6), issues to do with salaries and job contracts (6), opportunities for development and career progression (5), university bureaucracy (4), relationships with colleagues (2), students and classroom issues (4), and language (3). The vast majority of comments in the ‘work adjustment’ category were of a problematising nature. Whilst not necessarily expressing negative feelings or experiences, they did point to challenges or raised issues of concern. These were related mainly to job contracts and salaries (18 comments), the research landscape (15 comments), students and classroom issues (13), Thai culture and its impact on work (10), language issues (9), and relationships with colleagues (5). These key themes are further unpacked below supported by relevant data extracts to give ‘voice’ to participants.

#### The research landscape

Aspects of the research landscape in Thai HE were experienced as positive, in particular access to research funding which was perceived by the academics, by and large, as less competitive than in their countries of origin although there was also some criticism regarding how funding opportunities were communicated.

*‘It was very easy to apply for funding.’ (FG1, P2).*

*‘A lot of funding is nepotistic if you don’t know the person who’s the chair of the committee then you don’t even know the funds are available.’ (FG2, P4).*

Participants also emphasised that there seemed to be fewer restrictions and constraints on what to research and reported feeling ‘free’ to set their own research agendas.

*‘I was able to work out my ideas when coming to Thailand. It was very good academic freedom.’ (FG2, P2).
*

Whilst several participants described themselves as research-active and reported feeling productive, our data suggests that this was often due to sustained efforts by individual academics rather than because of a structured research support system more generally. A lack of institutional support for research activities, in particular where these were seen as being in conflict with teaching commitments, was identified by several participants. Whilst not necessarily set out explicitly in job contracts, there was a sense that host institutions generally wanted their international academics to be research-active but that support for research activities was mostly lacking. Some participants were actively discouraged by their institutions to pursue further research studies.

*‘They want research but they’re not willing to support it that heavily.’ (FG3, P2).*

*‘It’s kinda two faced in some ways…I don’t think it’s intentional deception but they want you to have the credentials they want you to be publishing and researching and going to conferences but they’re not willing to kind of support it all the time.’ (FG3, P2).*

*‘They want you to do something but they don’t want to pay you for it.’ (FG3, P4). *

Gaps in institutional support appeared to be filled by national funding sources and external research networks which were commented on positively.

*‘In my university itself there’s no support. I have support from other research networks inside Thailand who give me things like money.’ (FG3, P5).*

*‘The university don’t doesn’t fund too much but they they have some but yeah the TRF* 3 *it helps a lot.’ (FG1, P3).*

Those academics who pursued research activities described it as a largely isolated experience with few opportunities for collaborative research. Instigating and maintaining collaborative research relationships was described as challenging, partly due to apparent heavy teaching and administrative workloads for local Thai staff.

*‘It’s very hard for a collective researcher. All of my publications have been individual…the majority of them that they’re not into research and as well as the time when you have to teach and administrative work research requires a lot of hours and a lot of dedication that sometimes people do not have.’ (FG2, P1).*

There was also a sense that host institutions, in some instances, prioritised quantity over quality of publications in their promotion criteria.

*‘One of the biggest problems I think one one thing wrong with a lot of Thai promotion and so on is there’s excessive reliance put on numbers of publications right so that there’s not enough emphasis on quality of what’s being done there’s too much on just you know you counted 1 2 3 4 5 6 7 8 9 10 papers.’ (FG2, P3).*

#### Relationships with students and staff

A fair amount of discussion revolved around the nature of relationships with fellow staff and students at the host institutions. Where participants spoke about their teaching, the general classroom atmosphere was mostly described as ‘enjoyable’ with students being viewed as ‘hard working’ and ‘polite’. Comparisons were made between Thai student cohorts and those in participants’ home countries with the former generally being viewed more positively.

*‘Comparing it with Australia, I’d say the students here are much more polite.’ (FG2, P3).
*

There were, however, some concerns that participants highlighted in particular regarding the quality of student cohorts on their programmes and their ability to engage critically with academic material. The latter was perceived as largely lacking with several academics identifying this as problematic. Critical thinking, creativity and participation in classroom discussions were identified as areas that need development.

*‘I also enjoy their respect from the students but I would like them to ask me the questions not just wait until I leave the classroom.’ (FG1, P3).*

*‘In terms of politeness it’s very good in terms of critical thinking I think that is a weak point.’ (FG2, P1).*

Relationships with local Thai colleagues, particularly with those in international HE institutions who had spent a period of time abroad themselves, were mostly experienced as ‘well-functioning’ and positive. There were, however, some reports of a perceived separation between internationally mobile staff and their domestic colleagues, and several participants pointed to a ‘lack of mixing’ between the two groups. Some staff described instances of not being invited to social gatherings or department meetings, and of feeling excluded from university events such as graduation ceremonies. Language barriers were identified as one possible cause, but there was also a feeling of local staff not being proactive in seeking out contact with their international colleagues.

*‘Often they have activities such as lunch with big buffet in front and they do that openly in front of everyone meetings celebration probably Thai…we don’t even know but they stay together.’ (FG3, P6).*

*‘I’ve felt like a lot of institutional separation from the Thai side for example at like graduation it’s all in Thai and they don’t offer any translating and we’re expected to go and like sit there for three hours and smile pretty for the cameras because we’re the foreign faces to show off to parents.’ (FG3, P2).*

The latter comment was indicative of a general feeling that international staff were predominantly being used as figureheads to showcase how ‘international’ an institution was, without being necessarily integrated into the academic and social fabric of an institution. There was some speculation as to whether Thai host institution actually knew ‘what to do’ with their international staff which was reflected in a lack of clarity in expectations and responsibilities and, in some cases, even job titles (see below).

#### References to Thai ‘culture’

There were several references to Thai culture and how this might influence different aspects of academic work, as illustrated in the following exchange from FG3 about peer teaching observations.

- *P5: yeah I asked some people to come to my class to give me appraisal oh no no we can’t it like it’s almost like it’s not.*
- *P4: but that’s Thai traditional culture*
- *P2: yeah*
- *P4: we don’t judge one of your peers*

One participant emphasised this further by stating:

*‘There’s no there’s no distinction between critical evaluation and personal attack.’ (FG3, P5).*

Cultural traditions impacting the teacher-student relationship were discussed several times and perceptions of these varied, as shown in the exchange from FG1 below on wai khru^4^. It can be seen from this example that whilst aspects of certain Thai traditions and rituals were appreciated by the international academics, some also disagreed fundamentally with their underlying purpose.

- *TF2: and they walk on their knees from that [end of] the room carrying heavy you know bouquets [of flowers and then].*
- *TM1: [hierarchy hierarchy] hierarchy*
- *TF2: they have their [forehead] on the floor just giving these flowers to you that’s [very uncomfortable].*
- *TM1: [I find it] terrible (.) really*
- *TF2: [yeah yeah]*
- *TM4: I disagree with*
- *TM1: imposing the power of bureaucracy*
- *TM1: [on your knees] down there (.) I get a salary]*

‘Fitting into’ Thai culture was not always perceived as straightforward and participants recalled instances when they had made cultural blunders.

*‘There is a long long period of learning…I think I needed at least five years to understand it.’ (FG1, P1).*

#### Immigration bureaucracy

Immigration and visa processes were experienced by most participants as stressful, in particular having to renew work permits on an annual or biennial basis which resulted in a feeling of uncertainty and lack of permanence. Some were especially unhappy with the medical examination required as part of this work permit process.

*‘I don’t want give my blood to work and the other thing I’m quite insulted if I catch a disease here they they kick you out.’ (FG1, P4).*

*‘I would say that the work permit and visa all that sort of stuff that is for me that’s a nightmare.’ (FG1, P6).*

*‘I pay more than a hundred thousand Baht in income taxes a year still every semester I have to go to renew my visa and my work permit in an atmosphere that officially calls me not foreigner that calls me alien.’ (FG1, P1).*

There was a general sense that the bureaucracy required to work as an overseas employee was highly complex and that higher education institutions were seemingly often unaware of this. Some older participants were concerned about regulations that made obtaining a retirement visa difficult.

*‘On the bureaucratic side of things I don’t think that the institutions or the government understand what it takes to get a foreigner to come to Thailand they there is not an easy path to stay here and to retire here.’ (FG1, P4).*

The reporting system required from overseas workers, both by the host institution and the national immigration authorities, was also experienced as restrictive.

*‘I cannot just leave the country without telling the university because of this stamp on my passport.’ (FG1, P3).*

*‘I’m a felon for failing to report.’ (FG1, P5).*

#### Working conditions

Several participants described their salaries as ‘low’, especially when supporting a family.

*‘Teaching is not a well paid job in Thailand.’ (FG1, P6).*

For some of the older participants, financial security in older age was also identified as an issue.

*‘It’s a little bit hard to save for retirement considering that the in my case the university doesn’t pay into any sort of retirement package for us.’ (FG2, P5).*

It was not uncommon for academic staff to take on extra evening or weekend work to make ends meet. Whilst most appreciated opportunities for extra work, such as additional English language teaching and invigilating exams, they also reported a lack of clarity around salaries for this type of casual work and problems with getting paid their wages on time.

*‘It was easy to make extra money…there were some months that I barely touched my salary.’ (FG1, P2).*

*‘They wouldn’t even tell you tell me what I’m getting paid per hour on the weekend until they paid me and they wouldn’t pay me for ten months.’ (FG1, P4).*

*‘The wage that I was offered was immensely low and in order just to get a normal wage I had to work on Saturday and Sunday.’ (FG1, P4).*

Most of the interviewees were on short-term or rolling contracts which were experienced as precarious and identified by some as a barrier to career advancement. These conditions were also blamed for a perceived high staff turnover rate and apparent lack of retention of international academics.

*‘It’s a yearly contract there is no career progression.’ (FG1, P5).*

*‘For sure my contract will not be renewed I don’t have any right like like that and so there is a lot of uncertainty and about retirement as well.’ (FG2, P5).*

It was not uncommon for participants to express feelings of uncertainty in this regard:

*‘Lecturers now since I think I think 2010 or so get only semester long contracts so I do the visa run and and the work permit run twice a year or I never know whether I will get a contract next semester.’ (FG1, P1).*

Several staff reported a lack of clarity around their roles and responsibilities at work and, at times, even in relation to their actual job title. Other participants reported confusion on the part of the institution in terms of expectations of overseas academics.

*‘I don’t think they know what they want us to do.’ (FG1, P2).*

*‘It’s not even been specified so I think that it might be expert or foreign expert.’ (FG3, P5).*

*‘It’s not obvious to me sometimes I would like to know what what is it they expect of me other than what it says in my contract.’ (FG3, P5).*

University bureaucracy, in particular committee work and paperwork associated with teaching, was experienced by many participants as overwhelming.

*‘We kind of joke in our department that there’s paperwork for paperwork…I have never experienced the level of bureaucracy that I have compared to here in Thailand.’ (FG3, P2).*

Several participants also pointed to a lack of opportunities for professional development and career progression.

*‘If people are looking for promotion and career moves and things like that I doubt whether that’s gonna really come about here.’ (FG1, P6).*

* ‘For personal development it’s very hard I find.’ (FG2, P5).*

#### Language issues

One barrier that seemingly prevented international staff from participating fully in the university community was to do with language proficiency, or lack thereof. Very few participants described themselves as fluent in Thai and many university meetings and events were reportedly not translated into English. Where translation was offered this tended to be done on an ad hoc basis or had to be pursued actively by international staff themselves.

*‘Even mandatory meetings that we’ve had a lot of time that are with the whole university are either haphazardly translated not translated or important documents we’ve needed we’ve had to really pursue translation for.’ (FG3, P2).*

*‘I get lots of information every day and I can never understand any of it.’ (FG3, P3).*

*‘I feel out of definitely feel out of kind of loop of sort of things that are going on.’ (FG3, P5).*

Some staff acknowledged explicitly the importance of learning the local language in order to fully participate in the university community.

*‘I think for me I would have had a different experience if I really worked hard at learning Thai because everything was coloured by the lack of Thai language. I felt very excluded from the Thai meetings and the training.’ (FG1, P2).*

There was a sense of language barriers impacting on opportunities for professional development and career progression.

*‘…most of the things that happen at the university level would impact on us we don’t really become part of the process so we we usually just get told this is what’s going to happen now you…and we (.) we I never get to attend trainings I was stagnant.’ (FG1, P2).*

Participants had to actively seek our opportunities for language learning with some paying for external classes and private one-to-one tutoring. Very little language support was seemingly provided by host institutions. There was also a perception that there was little awareness amongst host institutions of the importance of Thai language skills for incoming academics.

*‘…they’re not very supportive I mean they did I didn’t think they’re aware that foreign teachers really need Thai language.’ (FG1, P2).*

## Discussion and Conclusion

Our focus in this study was on the human experience of ‘internationalisation’, rather than on HE internationalisation as a numbers-driven phenomenon (see Young, 2016; Schartner & Young, 2020). Our analytical focus was on the idea of adjustment, specifically in and across the intersecting domains of psychological, sociocultural and professional/work adjustment. We found that elements of the former two captured some of the experiences described, the latter, particularly where it intersected with the sociocultural, were the most successful in capturing the experience of our sample of international academic staff working in Thailand. We therefore conclude that in this respect at least, the conceptual framework can serve as a useful heuristic beyond the groups investigated to provide its original empirical basis – ‘international’ postgraduate students at a UK university – and for both staff and for non-nationals working or studying in universities in the global South (e.g. Srisakda, 2018).

Most of the findings served to confirm, but also perhaps to deepen understandings of, migration to and within HE in the global South. This is particularly true of course in respect of Thailand, with an increasingly large and important non-national academic workforce and a policy-driven aspiration to be ‘more international’ and ‘successful’ – in recruitment terms - in its HE sector. The experiences of ‘international’ academic staff in Thailand, as with much of the global South, has received very little research attention, as we note above. We hope and believe that the research detailed here provides important benchmarking information for governments and for HE sectors as to how (and perhaps how not) to make the best out of the challenges and opportunities presented by a diverse staff cohort if internationalisation as a lived phenomenon is to be made more positive. A committed, involved and to some extent settled community of non-national academic staff clearly have a lot to give to help realise recruitment and retention aspirations.

Our findings, however, indicated strongly that for many such academic staff, the experience was, on balance, far from uniformly positive. We found for example, in line with earlier research by Snodin et al. (2021), Richardson and McKenna (2006) and Hoy (2009) a sense among the majority of our participants of distancing, even exclusion, from the host ‘professional culture’. The role of language competence, or a lack of it in respect of Thai, seemed crucially related to a sense of belonging and being able to fully participate professionally. Such a finding aligns with much of the recent literature exploring ‘expat’ adjustment, and particularly academics’ adjustment, which highlighted the importance of facility in a/the local language or languages to successful adjustment (e.g. Young et al., 2013). This is true even in professional environments where a Lingua Franca predominates, as with English on most ‘international’ programmes of study, an increasingly advertised feature of the ‘offer’ of Thai HEIs. Some responsibility for acquiring the necessary competences clearly lies with academic migrants themselves, but it also behoves HEIs to offer language help and support in this respect – on the evidence of our study, not enough support of sufficiently high quality is being offered. This extends, we contend, to offering lingua-*cultural* training to facilitate adjustment, particularly for those who might experience a more ‘culturally-distanced’ adjustment (in Isakovic and Forseth Whitman’s 2013 conceptualisation) to facilitate understanding of and involvement in local professional cultures. This may also serve to reduce any perceived threat migrants make to Thai local culture, values and security if intercultural dialogue is at the heart of such understandings (Burford et al., 2019).

A number of avenues for future investigation are suggested by our findings. The nature of recruitment practices among Thai universities, and the factors that determine the selection of international staff clearly need bringing out into the open. Support currently given to non-national staff by Thai HEIs, and the sharing of good practice in this regard, should be explored and encouraged. The immigration and work-place status and job-security for non-national staff are likely to involve complex discussions about the needs of Thai HE and of wider society in Thailand and how these needs align with Thailand’s immigration policies and employment practices, particularly practices related to ‘international experts’. Such discussions are urgently needed if Thailand wants and needs a reasonably settled and fully-committed international academic workforce to help it to achieve its aspirations in terms of HE.

## Data Availability

Not applicable.

## Footnotes

1 Sanuk is a wide-reaching idea that embodies the playfulnsess and sense of humor that is so central to life in Thailand. (source: https://www.reachtoteachrecruiting.com/guides/thailand/culture-in-thailand/ accessed 26/Feb/2018)

2 Thai greeting – slight bow with palms pressed together in prayer-like fashion.

3 Thailand Research Fund.

4 Thai ritual where students show respect to their teacher.
